# Ultra-Sensitive and Specific Detection of *S. aureus* Bacterial Cultures Using an Oligonucleotide Probe Integrated in a Lateral Flow-Based Device

**DOI:** 10.3390/diagnostics11112022

**Published:** 2021-10-31

**Authors:** Isabel Machado, Garazi Goikoetxea, Enara Alday, Tania Jiménez, Xabier Arias-Moreno, Frank J. Hernandez, Luiza I. Hernandez

**Affiliations:** 1 SOMAprobes S.L., Mikeletegi Pasealekua, 83, 20009 Donostia, Spain; isabel.machado@somaprobes.com (I.M.); garazi.goikoetxea@somaprobes.com (G.G.); enara.alday@somaprobes.com (E.A.); tania.jimenez@somaprobes.com (T.J.); xabier.arias@gmail.com (X.A.-M.); 2 Department of Cellular Biology and Histology, Faculty of Medicine and Odontology, University of the Basque Country (UPV/EHU), 48940 Leioa, Spain; 3 Department of Physics, Chemistry and Biology, Linköping University, 58185 Linköping, Sweden; frank.hernandez@liu.se; 4 Wallenberg Centre for Molecular Medicine, Linköping University, 58185 Linköping, Sweden

**Keywords:** *S. aureus*, detection, lateral flow strips, nucleic acid probe, nuclease activity

## Abstract

The identification of pathogens causing infectious diseases is still based on laborious and time-consuming techniques. Therefore, there is an urgent need for the development of novel methods and devices that can considerably reduce detection times, allowing the health professionals to administer the right treatment at the right time. Lateral flow-based systems provide fast, cheap and easy to use alternatives for diagnosis. Herein, we report on a lateral flow approach for specifically detecting *S. aureus* bacteria within 6 h.

## 1. Introduction

Emerging pathogens that cause severe infections and diseases present an urgent need for the development of novel, more rapid and sensitive methods and devices that allow for timely patient management. Despite continuous efforts encouraged by governments and health institutions worldwide for the development of point-of-care (PoC) diagnostic tools that allow the simultaneous detection and identification of pathogens, there is still no test that combines all advantages and attributes for a test of this type [1,2]. Traditional microbiology techniques used in the identification of infections and causative bacterial species always start by culturing the samples, followed by several molecular and biochemical tests, such as colony size and color, Gram stain, catalase/oxidase activity, coagulase test, etc. These techniques are precise and effective; however, they are time consuming, provide delayed information (2 to 3 days), and are expensive due to the need of trained personnel and specific lab equipment. Nowadays, PCR-based approaches and matrix-assisted laser desorption-ionization time of flight mass spectrometry (MALDI-TOF MS) methods are being used for their speed and sensitivity [3,4,5,6,7,8,9]. However, amplification methods like PCR suffer from contamination problems, difficult experimental design, complex interpretation of results, and high costs. In the case of MALDI-TOF MS, the main limitation, besides the obvious high capital investment needed, is the inability to differentiate taxonomically related bacteria like high pathogenic species (e.g., *Shigella*) from commensal common bacteria (e.g., *E. coli*) and the need of an existing database that correlates the spectra with the pathogen [7]. As a result, these techniques currently co-exist with bacterial cultures and a diagnostic result is obtained after 18–24 h or even longer [8].

Consequently, there is an urgent and unmet clinical need for innovative PoC diagnostic tools that can reliably identify bacterial infections with short turnaround time, enabling doctors to treat patients with appropriate antibiotics as early as possible and to avoid the spread of bacteria and development of antibiotic resistance, especially in the hospital setting. The development of such rapid diagnostic methods will provide clinical and cost-related improvements in the management of infections.

Desirable features for the development of diagnostic devices should include simplicity of design, cost-effectiveness, portability, ease-of-use, no need for specialized personnel or complex laboratory instrumentation and short turn-around of results with high sensitivity and specificity. In the last decade, immunoassays have found wider acceptance due to their affordability, user friendliness and versatility of detection formats, making them an excellent choice for point-of-care applications [10,11]. Among all systems using antibodies as recognition molecules, lateral flow immuno-chromatography (LFIC) had recently achieved remarkably high level of impact, as it can be used for applications where fast and accurate detection is required [12,13], such as in the case of antibody or antigen tests for COVID-19. LFIC is a simple method of specifically detecting the presence or absence of a target analyte in a given sample matrix. LFIC is currently being used as a PoC tool (fast and in situ) in a large range of applications, from the widely known home pregnancy test to more specialized tests for clinical, food safety, environmental and industrial applications, eliminating the need for specialized and costly equipment and conventional laboratories [14].

In our lab, we have taken advantage of the features of LFIC, and we sought to combine them with our extensive expertise in harnessing the biology of bacterial nucleases as biomarker of disease to create an approach that provides rapid and sensitive identification of bacterial infections. Bacteria are microorganisms that express a variety of enzymes and proteins which are unique for each species, even for members belonging to the same genus, thus making them useful tools for specific targeting. Nucleases are one type of these enzymes, responsible for many functions related to the regulation of bacteria genetic material by cleaving nucleic acids, such as DNA or RNA. Moreover, nucleic acids have been proven to be useful recognition molecules for the development of several diagnostic strategies, mostly due to their flexibility and adaptability to various transduction mechanisms, such as fluorescence, electrochemical, piezoelectric, and colorimetric [15,16]. In recent years, we have been working on various approaches based on nucleic acid substrates to target specific nucleases that could be used as a blueprint of bacterial infections or specific types of cancer [17,18,19,20]. The *Staphylococcus aureus* (*S. aureus*) bacteria, both the methicillin sensitive (MSSA) and methicillin resistant (MRSA) varieties, is the leading cause of nosocomial infections worldwide [21,22,23,24]. Therefore, fast, accurate identification of this bacteria is crucial to ensuring effective treatment and preventing the spread of infections within hospital settings.

In a previous report, *S. aureus* infections were detected using an oligonucleotide fluorescent probe (TT probe) as a substrate that is specifically cleaved by the micrococcal nuclease (MN) produced by *S. aureus* in less than 1 h [20]. Herein, we propose to follow up on that study by translating this technology, based on nucleases and short nucleic acid (oligonucleotides) substrates, into an approach amenable to a portable and easy-to-use detection device based on a lateral flow system.

**Figure 1:**
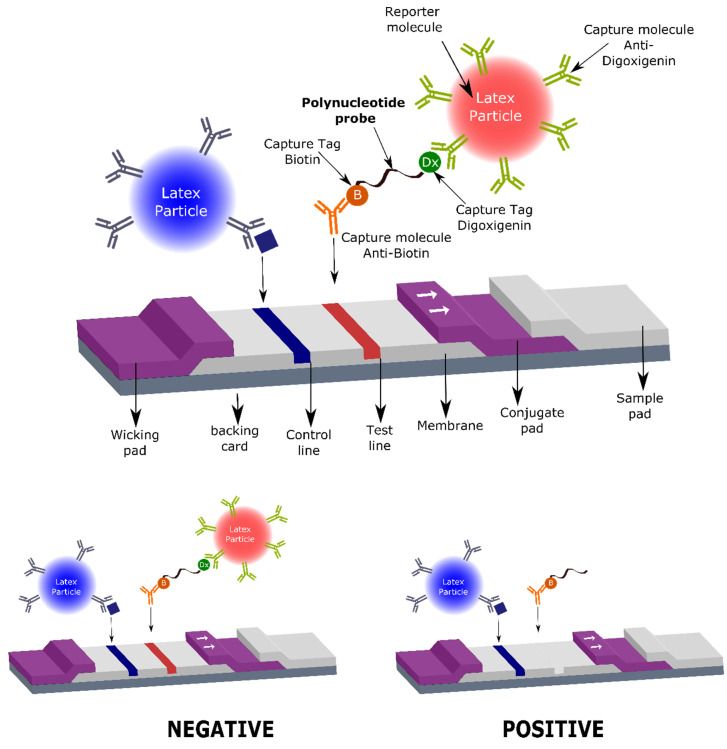
Schematic representation of the detection technology based on the activity of nucleases on oligonucleotide substrate probes, and its integration in a lateral flow assay.

Therefore, herein we describe, for the first time, the utility of specific oligonucleotide probes, modified with tags that allow their capture on a lateral flow strip, for the rapid, sensitive, and specific detection of nuclease activity derived from *S. aureus*, with a great potential for use in the clinical field. Specifically, the oligonucleotide probe that acts as substrate for the target nuclease is composed of a short oligonucleotide sequence flanked by two capture tags, digoxigenin and biotin (polynucleotide probe, Figure 1), and we named it the LF-TTprobe. This oligonucleotide sequence consists of 2 central thymidines as the specific region, flanked by nucleosides modified with a methyl group at the 2′-position of the sugar ribose (2′-O methyl), that renders the probe resistant to non-specific nucleases.

The digoxigenin tag (Dx) binds a reporter molecule (latex microparticle, red), previously functionalized with anti-digoxigenin antibodies, which resides in the conjugate pad of the strip. On the other hand, the anti-biotin molecule captures the nucleic acid probe in the test line of the strip by the other end, where the biotin (B) is attached. If the target nuclease (e.g., *S. aureus* nuclease) is absent, the detection complex formed by the nucleic acid probe and the red latex microparticle is visualized as a red line by accumulation of the red latex particle complexes in the test line area (negative result, Figure 1 lower left panel). If the target nuclease (e.g., *S. aureus* nuclease) is present, the nucleic acid probe is cleaved, releasing the oligonucleotide fragment along with the digoxigenin tag which is complexed to the red latex microparticle. In this situation, no test line is detected, thus indicating the presence of nucleases in the biological sample. Consequently, a positive result in this system shows no test line (Figure 1, lower right panel).

The LFIC system performance was tested using culture supernatants of *S. aureus* as the target pathogen, and culture supernatants of *Staphylococcus epidermidis* (*S. epidermidis*) as the control pathogen. The choice of the control bacterium was dictated by two factors. On one hand, *S. epidermidis* is the main bacterium present in clinical samples that might show cross-reactivity with *S. aureus*. On the other hand, both bacteria are endowed with similar surface-expressed proteins and secrete virulence factors such as toxins, and enzymes such as nucleases. However, when compared to *S. aureus*, *S. epidermidis* presents a smaller repertoire of nucleases [25], thus enabling technologies based on nucleases to discriminate between them. Our technology, presented herein, is based on harnessing the activity of a specific secreted nuclease, allowing for their differentiation. Moreover, both *S. aureus* and *S. epidermidis* are also clearly differentiated from other common bacteria causing severe infections, such as *Klebsiella pneumoniae*, *Pseudomonas aeruginosa*, *Escherichia coli* and *Streptococcus pneumoniae*, showing the high specificity of the method.

In this contribution, we demonstrate a significant improvement in the sensitivity of *S. aureus* detection when compared to the previously described fluorescent approach by achieving a 250-fold reduction in the amount of the oligonucleotide probe used for the optimal detection of its target nuclease, hence decreasing the potential cost of LFIC device manufacturing. Importantly, we show that we can detect the presence of *S. aureus* via its secreted nuclease activity in mini cultures of 6 h, obtained from one colony forming unit (CFU) culture, thus significantly shortening the actual waiting time of the gold standard method, which is usually between 18 to 24 h. This unprecedented three-in-one feature, increased sensitivity (S), specificity (S) and speed (S), has paramount implications on the development of LF applications for clinical diagnostics, with great potential to be extended to other pathogens and human conditions.

## 2. Materials and Methods

### 2.1. Buffer Solutions and Culture Media

Tris-EDTA, 1X solution (TE, CAS number: 38641-82-6, pH 8.0) was purchased from Thermo Fisher Scientific (Madrid, Spain). Tris buffered saline (TBS) purchased from Merck Life Science (Madrid, Spain) was prepared by dissolving the powder of a pHast pack in 500 mL of ultrapure water to obtain a solution of 1× with final concentrations of 50 mM Tris, 138 mM NaCl, 2.7 mM KCl, pH 8.0. Tween 20 was purchased from Sigma-Aldrich and used as received to prepare the running buffers for the lateral flow strips at a final concentration of 0.05% *V*/*V*. Calcium chloride (CAS number: 1043-52-4) purchased from Merck Life Science (Madrid, Spain) was prepared in ultrapure water at a final concentration of 100 mM. Phosphate buffered saline 10× (PBS, pH 7.4) without MgCl_2_ and CaCl_2_ (PBS-/-) was purchased from Thermo Fisher Scientific (Madrid, Spain). This solution was used at 1× by diluting it ten times in ultrapure water. Ethylenediamine tetraacetate acid (EDTA) 500 mL, 0.5 M, was purchased from Thermo Fisher Scientific (Madrid, Spain). PBS-/- with EDTA solution was prepared using PBS-/- 1× and EDTA 500 mM for a final concentration of 10 mM EDTA.

Tryptic soy broth (TSB), nutrient broth (NB), Todd Hewitt broth (THB) culture media, yeast extract and European bacteriological agar were purchased from Laboratorios Conda SL (Madrid, Spain) and prepared in ultrapure water, as indicated. The agar was always used by mixing 15 g per liter prepared with the broth for incubation in Petri dishes. All the media were always autoclaved at 121 °C for 25 min.

### 2.2. Bacterial Strains and 24 h Supernatant Preparation

All the bacterial strains used in this study were obtained from LGC Standards (Barcelona, Spain) and cultured using the culture conditions recommended by the American Type Culture Collection (ATCC). Table 1 details all bacteria strains used herein, with their respective culture media.

To prepare pure culture supernatants, frozen bacteria stored at −80 °C were pre-cultured in each correspondent medium (Table 1) for 18 h at 37 °C with agitation at 180 rpm. In the case of *Streptococcus pneumoniae,* the culture was always supplemented with 5% CO_2_. One milliliter of each bacterium in glycerol solution was added to 50 mL of the correspondent broth medium. After 18 h of growth, a dilution of 1:500 was prepared by adding 100 μL of the 18 h pre-culture to 50 mL of fresh medium (sub-culture). Using 10 μL disposable loops, Petri dishes with the corresponding agar were also prepared to obtain isolated colonies. Both, broth and dish preparations were incubated for 24 h at 37 °C with and without agitation at 180 rpm, respectively.

After 24 h of growth, each culture was then centrifuged (6000× *g* for 20 min) and the supernatant was removed and stored at −20 °C until use. The pellets were properly discarded in biohazard waste. The Petri dishes were stored at 4 °C until use.

### 2.3. Oligonucleotide Probes Synthesis and Purification

The probe was synthesized and purified at Biomers.net (Ulm, Germany). The oligonucleotide sequence of TTprobe was the following: 5′-mCmUmCmGdTdTmCmGmUmUmC-3′ (m = -methyl at 2′; d = DNA nucleoside with no modification). The TTprobe for the lateral flow tests contained a digoxigenin molecule at the 5′-end and a biotin molecule at the 3′-end (LF-TTprobe). For this, a standard method of solid phase phosphoramidite chemistry was used, followed by high-performance liquid chromatography (HPLC) purification. The probe identity was confirmed by MALDI-MS. The purity of the probe, as assessed by HPLC analysis, is typically greater than 95%.

The TTprobe used in the fluorescence experiments (F-TTprobe) was synthetized with a fluorophore and a quencher, as previously reported [20].

### 2.4. Oligonucleotide Probes Solution Preparation

The oligonucleotide probe stock solutions were prepared by resuspending the lyophilized probe vial (from the manufacturer) in TE buffer at a final concentration of 500 pmol·μL^−1^. Then, as needed, working solutions of the fluorescent probe were prepared by dissolving the F-TTprobe in PBS-/-. To prepare working solutions of the lateral flow probe, the LF-TTprobe was resuspended in ultrapure water. Note that probe solutions prepared in PBS are not recommended for lateral flow assays.

### 2.5. Lateral Flow Strips

The lateral flow strips used throughout this work were purchased from Operon (Cuarte de Huerva, Zaragoza, Spain) and Abingdon Health (Sand Hutton, York, UK). The Operon strips use latex particles as reporter molecules, while Abingdon PCR-Flex strips use carbon particles as reporters. Both strips contain anti-digoxigenin/biotin antibodies.

#### 2.5.1. Optimization of the Strips—Running Buffer

To better visualize the strip lines for an accurate reading of the lateral flow (LFA) results, an appropriate running buffer should be used. The running buffer should not only maintain the sample pH, but also allow a perfect flow through the strip, and not interfere with the antigen–antibody interactions. Therefore, we tested and evaluated the performance of two buffers: TBS buffer alone (Buffer 1) and TBS buffer supplemented with tween 20 0.05% *v/v* (Buffer 2).

#### 2.5.2. Optimization of the Strips—Probe Concentration

To determine the best concentration of probe to be used in the LF system, a titration with different concentrations of the LF-TTprobe was performed, using *S. aureus* ATCC 29213 supernatants as the sample and TSB medium and the *S. epidermidis* ATCC 35984 supernatant as the control. LF-TTprobe solutions at different concentrations (500, 300, 250, 200, 150, 100, 75, 50 and 5 fmol·μL^−1^) were prepared in ultrapure water.

### 2.6. Nuclease Activity Assays (NAA)

#### 2.6.1. Fluorescence Detection

The fluorescence nuclease activity assays were performed in reaction volumes of 10 μL, by mixing 8 μL of sample (24 h culture supernatants or control medium) with 1 μL of CaCl_2_ 100 mM and 1 μL of F-TTprobe (50 pmol·μL^−1^). The reactions were incubated at 37 °C, for 30 min. Then, the reactions were stopped by adding 295 mL of PBS-/- supplemented with 10 mM EDTA. Finally, 95 mL of each sample was loaded in triplicate into 96-well black plates (96F non-treated black microwell plate, Thermo Scientific). Fluorescence intensity was measured with a fluorescence microplate reader, Synergy Neo2 Hybrid Multi-Mode Reader from Agilent BioTek (Winooski, VT, USA) using the filter settings for FAM (excitation/emission 485/528 nm). Three independent experiments were performed with each sample. The results are expressed as the mean ± SD (*n* = 3) of fluorescence intensity in arbitrary units (a.u.).

#### 2.6.2. Lateral Flow Detection

The nuclease activity assays for the lateral flow detection were prepared in a similar fashion as for the fluorescence assays, by only changing the probe and probe concentration. Therefore, 8 μL of each sample (24 h culture supernatants or control medium), were mixed with 1 μL of CaCl_2_ 100 mM and 1 μL of LF-TTprobe (200 fmol·μL^−1^), to a final reaction volume of 10 μL. Then, the reactions were incubated at 37 °C for 30 min. Next, to allow the reaction to flow through the strip, 150 μL of TBS + tween 20 0.05% running buffer were added to the reaction. Thus, a final volume of 160 μL was transferred to a 96-well plate with a flat bottom so toallow the strips, when dipped in the wells, to be in contact with the entire volume of the sample. Then the strips were labeled, dipped in the correspondent well and the flow was allowed to run for 10 min. Next, the strips were removed from the wells and the results were visually read. Photographs of the strips were also taken and recorded.

#### 2.6.3. MALDI-TOF

For an optimal MALDI evaluation, the NAA was slightly modified from the original version. Thus, 1 μL of sample, *S. aureus* ATCC 29213, *S. epidermidis* ATCC 35984 or TSB as control, was mixed with 1 μL of CaCl_2_ 100 mM and 1 μL of LF-TTprobe (with digoxigenin and biotin) and prepared in ultrapure water at a concentration of 50 pmol·μL^−1^ (final concentration 5 pmol·μL^−1^) in 7 μL of ultrapure water and incubated at 37 °C for 30 min. Next, the reaction was not stopped, but was directly used for crystallization. The sample preparation was carried out by the deposition of 0.5 μL of the sample (NAA reaction) directly onto a polished stainless-steel plate (Bruker Daltonics, Bremen, Germany), and mixing it with 0.5 μL of matrix solution, which was composed of a dissolution of 10 mg of 2,4,6-trihydroxyacetophenone (THAP) and 5 mg of diammonium citrate in 1 mL of deionized water. MALDI-TOF MS analysis was performed using an UltrafleXtreme III time-of-flight mass spectrometer equipped with a Nd:YAG laser (Smartbeam II, 355 nm, 1 kHz), controlled by Flex Control 3.3 software (Bruker Daltonics, Bremen, Germany). The acquisitions were carried out in positive-ion linear mode at a laser frequency of 1 kHz. The spectrum was acquired at 30% laser fluency and was recorded in the *m*/*z* range from 1500 to 5000. The deflector cut-off was set at *m*/*z* 1300, and the spectrum resulted from the accumulation of 3000 laser shots. All the spectra were analyzed using FlexAnalysis software 3.0 (Bruker Daltonics, Bremen, Germany).

### 2.7. Sensitivity Tests

The sensitivity experiments were carried out using supernatants of *S. aureus* ATCC 29213 and *S. epidermidis* ATCC 35984. Tenfold serial dilutions, from 10^−1^ down to 10^−6^, of these supernatants were prepared in ultrapure water. Next, NAA for lateral flow was performed as previously described in Section 2.6.2.

### 2.8. Specificity Tests

The specificity experiments were carried out using supernatants of all bacteria detailed in Table 1, Section 2.2. The NAA for lateral flow was performed as previously described in Section 2.6.2.

### 2.9. Short Cultures of S. aureus ATCC 29213 and S. epidermidis ATCC 35984

Cultures of *S. aureus* ATCC 29213 and *S. epidermidis* ATCC 35984 were prepared from 1 CFU taken from previously prepared Petri dishes that were stored at 4 °C, as detailed in Section 2.2.

One CFU of each bacterium was added to 50 mL of TSB culture medium using a collection loop and incubated at 37 °C with 180 rpm agitation. Next, various samples were collected at the following incubation times: 2 h, 3 h, 4 h, 6 h, 8 h, 10 h and 12 h. One milliliter of each bacterial culture was collected and centrifuged at 13400 rpm for 20 min. The supernatants were then kept at 4 °C until use in the NAA. The pellets were properly discarded. Before proceeding with the NAA, all the samples to be used were prepared by mixing 10 μL of each sample with 10 μL of TBS buffer (1:1) (pre-NAA prep.). The NAAs were performed for fluorescence and lateral flow detection as explained in Section 2.6.1 and Section 2.6.2, using as a sample 8 μL of each pre-NAA prep.

## 3. Results and Discussion

### 3.1. Optimization of the Running Buffer

**Figure 2:**
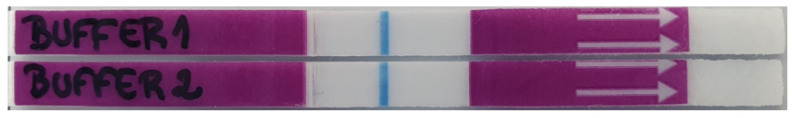
LF strips optimization. Selection of the running buffer. “Buffer 1” = TBS; “Buffer 2” = TBS + tween 20 (0.05% *v/v*).

The running buffer is an essential component of a lateral flow assay. A simple but well formulated running buffer allows for sample buffering by maintaining the pH constant, minimizing non-specific bindings, not interfering with the antigen–antibody interactions, and allowing the proper flow through the strip, thus helping to improve the signal intensity of the test line. Moreover, a simple buffer composition allows for easier preparation that can impact the reproducibility, shelf-life stability, and manufacturing quality of the final commercial product. In our system, the running buffer is added to the reaction volume after the nuclease activity on its substrate LF-TTprobe has had occurred, and thus the buffer needs to preserve the reaction products to be detected at the test line while avoiding interference with the antigen–antibody interactions. Therefore, we sought to optimize the LFIC system performance for the nuclease activity detection by electing the best running buffer (Figure 2). We have thus tested a standard TBS (pH 8.0) buffer (Buffer 1) which is generally used in lateral flow immunoassays, along with one additional buffer (Buffer 2) containing a small concentration of tween 20 detergent, which is also generally used in LF systems [26]. The role of the detergent is to facilitate the dragging of the elements along the strip and to provide a clean white background for better readings of the results. Figure 2 shows the excellent performance of both buffers by allowing a clean flow through the strips, resulting in very clear and well-defined control bands (blue lines).

As expected, since no probe was used in this experiment, no line developed at the test band. Notably, the test band zone is very clean, with no shadows or smudges (see Figure 2), hence no visible indication of non-specific interactions between the buffer and the strip’s elements, more specifically between the latex particles functionalized with the anti-digoxigenin antibody and the anti-biotin antibody.

### 3.2. Optimization of the Probe Concentration

**Figure 3:**
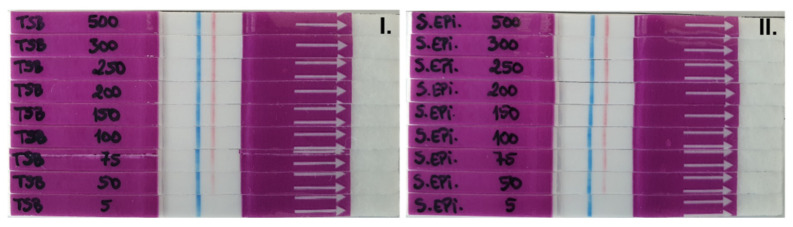

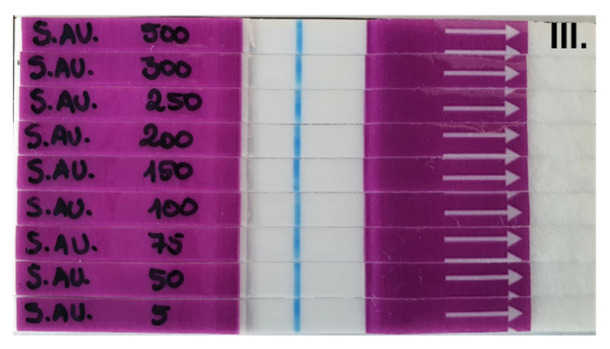
LF strip optimization. Determination of the optimal LF-TTprobe concentration. **I**. TSB and **II**. *S. epidermidis* ATCC 35984 were used as control medium and bacterium, respectively, allowing for determination of the limit of visible detection for both lines, as a negative result in our system. **III**. *S. aureus* ATCC 29213 was used as the target pathogen to obtain a positive result. The blue line represents the strip’s Control line, red line represents the strip’s Test line.

Next, we sought to optimize the concentration of the probe for detection in the LF system (Figure 3). As described in Figure 1, the readout in our system is based on the absence of line at the test band (Figure 1, lower right panel) for a positive result, and the presence of a line at the test band (red line) for a negative result (Figure 1, lower left panel), along with the control (blue) line for a valid result. We defined the optimal concentration of the LF-TTprobe by testing TSB culture medium as background or sample matrix control, while the supernatants of the 24 h culture of *S. epidermidis* ATCC 35984 (control bacteria) and *S. aureus* ATCC 29213 (target bacteria) were used as the negative and positive sample, respectively.

Having in mind the qualitative, binary readout of the LF system through either the presence or absence of a line, we sought to optimize the amount of the probe to increase the detection sensitivity. Thus, we have decreased the concentration of the LF-TTprobe from the picomole scale usually used in our fluorescent system to the femtomole scale for the LF system. Thus, we tested 8 dilutions of the probe, starting from 500 fmol·μL^−1^ down to 5 fmol·μL^−1^ (Figure 3). As seen in Figure 3I and II), the limit of detection for the LF-TTprobe reached by the system was 50 fmol/μL, the concentration at which the test line (red color) was no longer perfectly visible. To ensure robust and consistent visual readings for the negative results (well defined red lines), we have chosen 200 fmol·μL^−1^ as the optimal concentration for further experiments. Importantly, for all LF-TTprobe concentrations used, no line at the test band was detected for *S. aureus* samples (Figure 3III) meaning that full probe degradation was achieved by the *S. aureus* nuclease present in the supernatant. These results show the great performance of the system for detecting bacterial nucleases in culture. Moreover, we demonstrate the feasibility of technology transfer by modifying our previously developed fluorescent TTprobe so that is amenable to immunocolorimetric detection in a lateral flow system (LF-TTprobe).

### 3.3. LF-TT Probe Versatility

**Figure 4:**
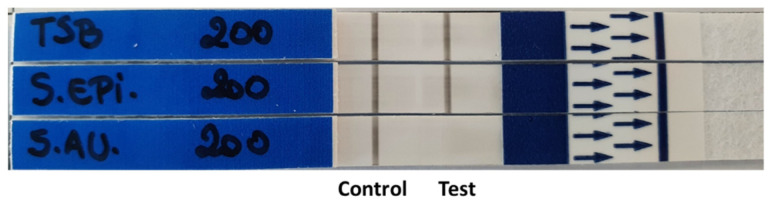
LF strip optimization. Versatility of the LF-TT probe for other commercially available strips containing a similar system.

Next, we sought to evaluate the versatility and robustness of the LF-TTprobe for detection in other similar lateral flow systems. To do so, the same samples used in Figure 3 were tested in LFA strips purchased from a different company (Abingdon Health, York, UK) using the optimized probe concentration of 200 fmol·μL^−1^. These strips use carbon particle conjugates; thus, the bands are visualized as black lines. Figure 4 shows the performance of the LF-TTprobe in these strips, with well-defined and visible lines (at control and test bands) for the TSB media and *S. epidermidis* as negative sample, while no line at the test band was observed for the *S. aureus* positive sample, showing complete degradation of the probe.

**Figure 5:**
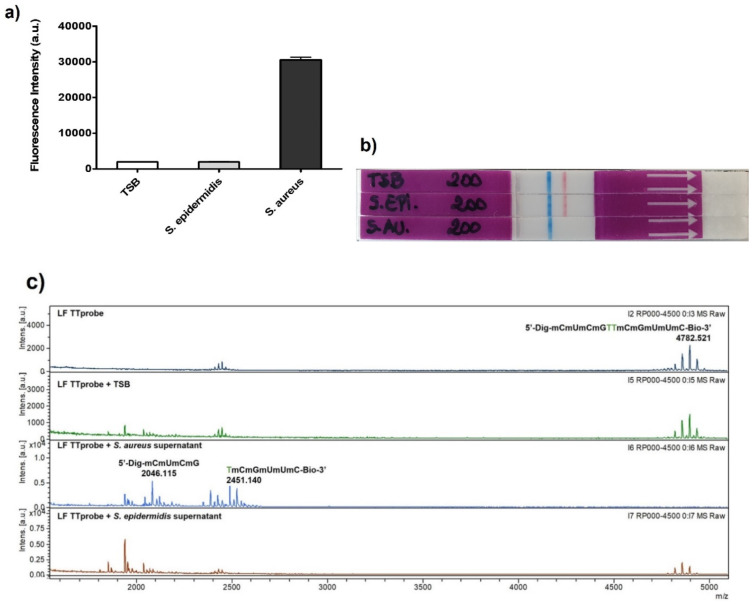
Comparison of three different techniques that prove the versatility of the TTprobe and its specificity for *S. aureus*. The cleavage of the TT probe by *S. aureus* was characterized by (**a**) fluorescence, where a significant increase in fluorescence intensity was detected, (**b**) lateral flow, proven by the absence of line at the test band, and (**c**) the MALDI-TOF MS spectrum of the digested and non-digested LF-TT probe.

To demonstrate that TTprobe is efficiently, specifically, and robustly degraded by the *S. aureus* nuclease, we compared three different detection methods, such as fluorescence (Figure 5a), lateral flow (Figure 5b) and MALDI (Figure 5c). For the fluorescence detection, the TTprobe was synthesized as previously described (see materials and methods section), with a fluorophore and quencher, thus the probe is initially in an OFF state. The methodology is based on a FRET system, where, in the presence of nuclease activity, the probe is degraded, and the fluorophore-containing sequence is released. Thus, an increase in fluorescence intensity can be detected (ON state).

Figure 5a shows a significant increase in nuclease activity, as given by the high fluorescence intensity, in the presence of *S. aureus* supernatants (black bar), while no increase in fluorescence intensity, and thus no nuclease activity, was observed for *S. epidermidis*, the non-target bacteria. In these experiments, a final concentration of 5 pmol·μL^−1^ of TTprobe was used for the nuclease activity reaction.

Fluorescence detection is a quantitative method that provides good sensitivity and reproducibility and is routinely used in research labs for assay development. However, the high fluorescence background usually arising from the sample matrix lowers the sensitivity of detection at low amounts of the target, and thus this method is deemed suboptimal for the early detection of bacteria in a clinical sample. Moreover, the relatively large-sized equipment and need for specialized personnel for assay performance and interpretation of results makes it less versatile for certain applications.

As described above, for the lateral flow detection, the TTprobe sequence was modified with end tags (digoxigenin and biotin) to allow a sandwich formation by binding to antibodies residing at the control and test bands in the lateral flow strip (Figure 1). While only a qualitative method, our LF-TTprobe system can achieve high sensitivity owing to the nature of nuclease activity detection based on the degradation of the substrate TTprobe. This degradation is an enzymatic reaction that acts as an intrinsic amplification module, where the limiting factor is the amount of substrate probe available for cleavage. In this scenario, one nuclease can degrade thousands of TTprobe molecules, resulting in signal increase, or in this case, the disappearance of the line in the test band. Therefore, it is counterintuitive that in order to achieve a clean, no-shadow test band, careful optimization of the probe concentration is essential, and this will greatly depend on the final target application. As seen in Figure 5b, the *S. aureus* supernatants containing the secreted MN nuclease, can be clearly and easily discriminated from *S. epidermidis* at 200 fmol (20 fmol final concentration) of LF-TTprobe, representing a significant 250-fold less amount of probe needed for the reaction when compared to the fluorescence system.

To confirm the specific cleavage of the LF-TTprobe by the *S. aureus* nuclease, we used MALDI-TOF MS to provide the structural information and probe fragmentation pattern. MALDI-TOF MS is a powerful analytical technique in which the sample is mixed with a matrix and ionized with a laser beam into charged molecules, and ratio of their mass to charge (m/z) is measured by determining the time it takes for the ions to travel to a detector at the end of a time-of-flight tube. It can be used as confirmatory tool of molecular identity in a wide range of applications and has recently emerged as a potential tool for microbial identification and diagnosis [27]. However, for the accurate identification of microbes, MALDI-TOF MS has its own drawbacks, by heavily relying on the database containing spectra of known organisms [7]. Herein, we used MALDI-TOF MS to corroborate the degradation efficiency of the LF-TTprobe with the fluorescence and lateral flow methods. To do so, the same samples used to generate Figure 5b, the TBS medium, *S. aureus* culture supernatant and *S. epidermidis* culture supernatant, were incubated with the probe for 1 h, and then the reactions were subjected to mass analysis.

Figure 5c depicts the fragmentation profile of the LF-TTprobe alone (grey spectrum), in TBS (green spectrum), in the *S. aureus* supernatant (blue spectrum) and in the *S. epidermidis* supernatant (brown spectrum). As observed from the top spectra of the LF-TTprobe only and in TBS media, the starting material is represented by a cluster of peaks at 4782.521 *m*/*z*. On the other hand, the TTprobe in the *S. aureus* supernatant shows a total shift of the peaks to the left side of the spectrum, with two clusters of peaks (blue spectrum) corresponding to the two major fragments of the cleavage, with masses compatible with the fragments Dig-5′-mCmUmCmG (2046.116 m/z) and dTmCmGmUmUmC-3′-Bio (2451.140 *m*/*z*). This fragmentation pattern implies the cleavage by a hydrolysis reaction of the phosphodiester bond at the 5′-thymidine position, being in very good agreement with our recently reported observations [28]. As expected, no fragmentation was observed for the LF-TTprobe in the *S. epidermidis* supernatant (bottom panel, brown spectrum).

### 3.4. Sensitivity and Specificity of the System

**Figure 6:**
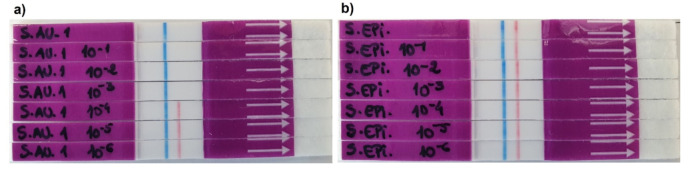
Sensitivity of the LF system with several dilutions of 24 h culture supernatants of (**a**) *S. aureus* ATCC 29213 (target bacteria), and (**b**) *S. epidermidis* ATCC 35984 (control bacteria). Presence of red test line indicates a negative result, and no red line indicates a positive result. The blue line represents the control line.

The sensitivity of the LF system was tested by performing 10-fold serial dilutions of the *S. aureus* and *S. epidermidis* culture supernatants. The nuclease activity assays were carried out for the 24 h cultures using undiluted and diluted (down to 10^−6^) samples of bacterial supernatants to determine the minimum dilution factor that allows the development of test line (red line) for the negative sample of *S. epidermidis* and the absence of the test line (no red test line) for the positive sample, *S. aureus*. Herein, the presence of *S. aureus* was detected down to a dilution of 10^−3^ (1:1000), representing the maximal dilution at which nuclease activity can be detected in culture supernatants of this bacterium (Figure 6a). For a higher dilution factor, the development of a well-defined red test line signifies that there was not enough micrococcal nuclease present in the sample to allow full digestion of the TTprobe. Therefore, at these low concentrations (10^−4^ to 10^−6^) the system was no longer able to detect *S. aureus*. For *S. epidermidis*, all dilutions tested resulted in well-defined red test lines, as expected (Figure 6b).

**Figure 7:**
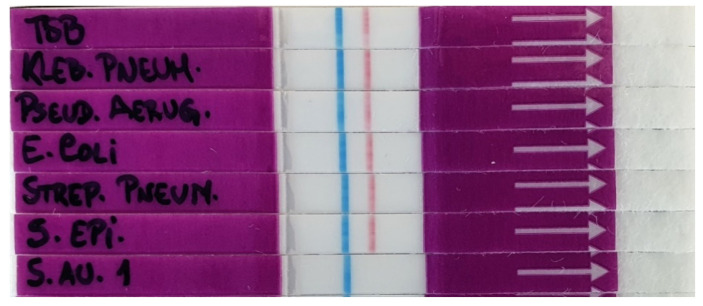
Specificity of the LF-TTprobe for *S. aureus* detection when tested with other common pathogens.

To study the specificity of the LF-TTprobe in the LF system, we tested culture supernatants of four non-Staphylococci bacteria (*Klebsiella pneumoniae*, *Pseudomonas aeruginosa*, *Escherichia coli* and *Streptococcus pneumoniae*) (Table 1) which, together with *S. aureus*, represent the most common pathogens causing community-acquired and nosocomial infections. TSB and *S. epidermidis* were used as the control medium and negative control Staphylococci bacterium, respectively. Figure 7 shows that all the non-Staphylococci bacteria, TSB, and *S. epidermidis* resulted in a well-defined red test line, meaning that none of these were able to digest the LF-TTprobe, thus proving the specificity of this probe for *S. aureus* detection. No red test line developed for the *S. aureus* strip, as expected for a positive result.

**Figure 8:**
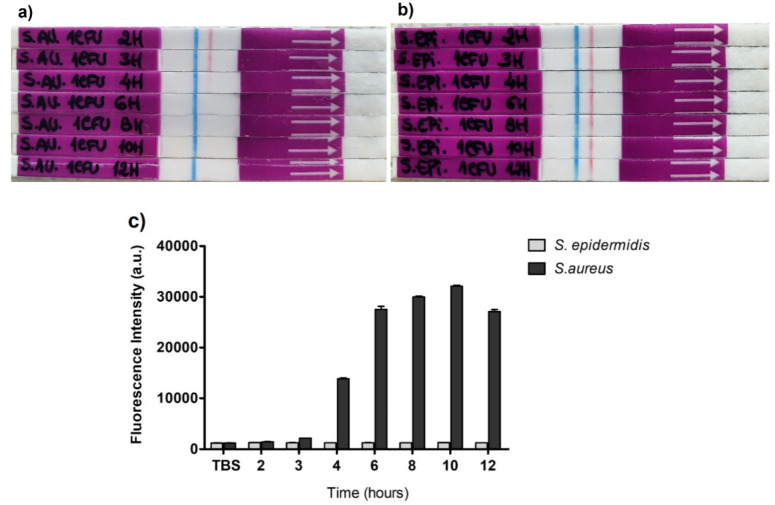
Detection of *S. aureus* ATCC 29213 in mini cultures by the lateral flow (**a**,**b**) and fluorescence (**c**) methods. The mini cultures were established from 1 CFU bacteria and NAA was performed at various times from 2 to 12 h. *S. epidermidis* was used as control bacteria. Error bars in **c**) represent the avg ± stdv. of at least 3 measurements.

We then sought to evaluate the sensitivity of the *S. aureus* detection in short-time cultures, which we called mini-cultures, of 2 to 12 h using both the LFA and fluorescence methods. Figure 8 shows the ability of both systems to detect the *S. aureus* nuclease in mini-cultures, as early as 6 h growth from 1 CFU bacteria. At this incubation time, and at later time points, complete degradation of the TTprobe occurred, resulting in a totally clear test line (Figure 8a, the fourth to eighth strips down) and high fluorescence intensity (Figure 8c, black bars). Interestingly, in the 4 h mini-culture, significant increase in fluorescence intensity can already be observed (Figure 8c, black bars) with nearly complete degradation of the probe, as also seen in the LF strip (Figure 8a—third strip down). For *S. epidermidis*, all samples tested resulted in well-defined red test lines, meaning no probe degradation, as expected (Figure 8b).

## 4. Conclusions

The main hurdle in the clinical diagnosis of infectious diseases remains the speed of pathogen identification. Despite the major technological advances in molecular diagnostics, digital microbiology and mass spectrometry, that have slowly found their way into microbiology laboratories, there still exists a global unmet need for diagnostic methods that are fast, accurate, simple and affordable. This need represents a real problem, being a life and death situation in underdeveloped regions with limited access to healthcare.

Herein we have developed and optimized a simple method for the detection of *S. aureus*, one of the major life-threatening pathogens, in mini cultures of only 6 h, representing a significant reduction of time when compared with the 18 to 24 h or longer of the standard methodologies. We have achieved this by transferring the detection of *S. aureus* nuclease (MN) from the previously reported FRET system to a lateral flow immuno-chromatography assay. Therefore, this system embodies the three most important features of a rapid diagnostic test for *S. aureus*, namely increased sensitivity (S), specificity (S) and speed (S), with high potential to be extended to other pathogens.

## Figures and Tables

**Table 1:** Bacterial type strains and the corresponding culture medium used.

Bacterial Strains	Medium
*Staphylococcus aureus* ATCC 29213	TSB
*Staphylococcus epidermidis* ATCC 35984	TSB
*Klebsiella pneumoniae* ATCC 13883	NB
*Pseudomonas aeruginosa* ATCC 10145	NB
*Escherichia coli* ATCC 25922	TSB
*Streptococcus pneumoniae* ATCC 49619	THB+yeast extract 2%
